# Tight intra-operative blood pressure control versus standard care for patients undergoing hip fracture repair – Hip Fracture Intervention Study for Prevention of Hypotension (HIP-HOP) trial: study protocol for a randomised controlled trial

**DOI:** 10.1186/s13063-017-2066-5

**Published:** 2017-07-25

**Authors:** Iain Keith Moppett, Stuart White, Richard Griffiths, Donal Buggy

**Affiliations:** 1 Anaesthesia and Critical Care Section, Division of Clinical Neuroscience, University of Nottingham, Nottingham, UK; 2 Department of Anaesthesia, Brighton and Sussex University Hospitals NHS Trust, Brighton, UK; 3 Department of Anaesthesia, Peterborough & Stamford Hospitals NHS Trust, Peterborough, UK; 4 School of Medicine, Mater Hospital, Eccles Street, Dublin 7, Ireland

**Keywords:** Humans, Hypotension/complications, Hypotension/mortality, Monitoring, Intra-operative, Post-operative complications/aetiology, Hip fractures/surgery, Acute kidney injury/aetiology, Arterial pressure

## Abstract

**Background:**

Hypotension during anaesthesia for hip fracture surgery is common. Recent data suggest that there is an association between the lowest intra-operative blood pressure and mortality, even when adjusted for co-morbidities. This is consistent with data derived from the wider surgical population, where magnitude and duration of hypotension are associated with mortality and peri-operative complications. However, there are no trial to data to support more aggressive blood pressure control.

**Methods/design:**

We are conducting a three-centre, randomised, double-blinded pilot study in three hospitals in the United Kingdom. The sample size will be 75 patients (25 from each centre). Randomisation will be done using computer-generated concealed tables. Both participants and investigators will be blinded to group allocation. Participants will be aged >70 years, cognitively intact (Abbreviated Mental Test Score 7 or greater), able to give informed consent and admitted directly through the emergency department with a fractured neck of the femur requiring operative repair. Patients randomised to tight blood pressure control or avoidance of intra-operative hypotension will receive active treatment as required to maintain both of the following: systolic arterial blood pressure >80% of baseline pre-operative value and mean arterial pressure >75 mmHg throughout. All participants will receive standard hospital care, including spinal or general anaesthesia, at the discretion of the clinical team. The primary outcome is a composite of the presence or absence of defined cardiovascular, renal and delirium morbidity within 7 days of surgery (myocardial injury, stroke, acute kidney injury, delirium). Secondary endpoints will include the defined individual morbidities, mortality, early mobility and discharge to usual residence.

**Discussion:**

This is a small-scale pilot study investigating the feasibility of a trial of tight intra-operative blood pressure control in a frail elderly patient group with known high morbidity and mortality. Positive findings will provide the basis for a larger-scale study.

**Trial registration:**

ISRCTN Registry identifier: ISRCTN89812075. Registered on 30 August 2016.

**Electronic supplementary material:**

The online version of this article (doi:10.1186/s13063-017-2066-5) contains supplementary material, which is available to authorized users.

## Background

Recently, observational data derived from 11,000 patients in the Anaesthesia Sprint Audit of Practice (ASAP) study [1, 2] have shown that regardless of anaesthetic technique or definition, hypotension during hip fracture surgery is very prevalent. Seventy-eight percent and 62% of patients receiving general and spinal anaesthesia, respectively, had a 30% or greater decrease in systolic blood pressure, whilst 40% and 22%, respectively, had mean arterial pressure (MAP) <55 mmHg. In a general surgical population, this MAP threshold is associated with significantly higher 30-day post-operative mortality and prolonged length of inpatient stay [3]. ASAP data support this observation [2]: 30-day post-operative mortality was significantly higher among patients whose MAP fell below 55 mmHg intra-operatively than among those whose lowest MAP remained above this threshold (6.0% vs 4.6%, *p* = 0.002).

However, it has been argued that 30-day mortality is an insensitive proxy outcome measure of anaesthetic intervention in hip fracture management. It occurs 30 days after the intervention and so will be affected by any number of other factors that are unrelated to anaesthesia [4]. Other recent large U.S. retrospective observational studies of general surgical patients have found significant associations between intra-operative hypotension (MAP <55 mmHg) and increased risk of cerebrovascular accident [5], acute kidney injury and myocardial injury [6, 7], suggesting a plausible causative link between anaesthesia-induced hypotension and early post-operative ‘ischaemic’ complications. Prospectively, strict blood pressure control as part of protocolised treatment aimed at maintaining cerebral oxygenation has been found to significantly reduce the incidence of mild and moderate post-operative cognitive dysfunction [8–10]. Myocardial injury and acute kidney injury [11, 12] after hip fracture are independently associated with poorer outcomes (increased mortality and institutionalisation, prolonged length of stay and return to mobility) among patients with hip fracture.

On the basis of this body of research, we hypothesise that intra-operative hypotension causes critical ischaemia/hypoperfusion of the brain, heart and kidneys in older, frailer patients with hip fracture who have co-morbidities, producing measurable post-operative alterations in organ function and resulting in poorer outcome, We propose a prospective, multicentre, randomised controlled trial comparing protocolised intra-operative control of blood pressure with standard treatment, using a composite of defined cardiovascular, renal and delirium outcomes within 7 days as the primary outcome and 30-day post-operative mortality, fitness for hospital discharge, return to baseline mobility level and return to independent living as secondary outcomes. This paper describes the protocol for an internal pilot feasibility study prior to the main trial.

## Methods/design

The trial protocol has been prepared and is reported in accordance with the Standard Protocol Items: Recommendations for Interventional Trials (SPIRIT) guidance [13, 14] (*see* Additional file 1: Figure S1, Additional file 2).

### Study objectives

#### Primary aims

We aim to complete an internal pilot feasibility study to test the hypothesis that tighter intra-operative control of blood pressure improves a composite outcome of presence or absence of defined cardiovascular, renal and delirium morbidity within 7 days of hip fracture surgery compared with standard treatment.

#### Secondary aims

Secondary aims are to discover whether tighter intra-operative control of blood pressure has beneficial effects on other measures of patient outcome and to provide data to support a full trial, including estimates of primary and secondary outcome rates/distribution, separation of blood pressure control between groups, and recruitment rates.

### Study design

This is a prospective, parallel-group, double-blind (participant and investigator), randomised controlled clinical trial. Three centres will participate. Recruitment commenced on 22 December 2016, and total recruitment is expected to take 12 months.

### Study setting

The study setting comprises three U.K. secondary hospitals providing acute care for patients with hip fracture. Each hospital admits over 400 patients with hip fracture each year.

### Randomisation and blinding

Randomisation (on a one-to-one basis) will be carried out via security-sealed, sequentially numbered opaque envelopes containing details of group allocation. The sequence generation and preparation of envelopes are done by an individual not involved in the trial. Randomisation is computer-generated in blocks of unequal size using a random seed known only to this individual. Randomisation is stratified by intended mode of anaesthesia (spinal vs general anaesthesia) and Nottingham Hip Fracture Score (NHFS) (≤4 vs ≥5) [15]. The sequence is not revealed until data are locked. Participants will be randomised in the anaesthesia room immediately prior to induction of anaesthesia by the attending anaesthetist.

The investigators, patients, nurses and data-collecting staff will all be blinded to treatment allocation. The attending anaesthetist will not be blinded to treatment allocation.

Assessment of mobility will be made by the ward physiotherapists treating the patient. Patients are declared medically fit for discharge by the multi-professional team when all are satisfied that the participant has no ongoing needs for acute hospital care. This team is blinded to participant allocation.

### Selection of participants

#### Recruitment

Participants will be identified by emergency department staff, trauma co-ordinator nurses and the orthopaedic and anaesthesia clinical teams, which will then inform a member of the research team about the presence of the patient. Once the patient is identified, a member of the research team will approach the patient, conduct initial screening and take consent from of the patient. Active participation in the study will be until the 30-day follow-up phone call.

Participants will be informed that participation is voluntary and that they are free to withdraw at any time without affecting their care. Data on time to discharge and mortality are routinely collected for all patients with hip fracture at each institution, and participation in the trial will involve consent for this data to be used. Patients unable to provide informed consent will be excluded from the trial. Patients for whom language will be a barrier will also be excluded, owing to the small-scale nature of the study; patient information will not be available in languages other than English. In practice, non-English speakers represent a very small number of patients in this population group.

#### Inclusion criteria

- Primary hip fracture listed for surgical repair
- Aged >70 years
- Able to give informed consent

#### Exclusion criteria

- Any condition or impairment which, in the view of the investigator, would prohibit the patient from full participation in the study

### Standard care

Standard care will be identical in both groups. Only the control of intra-operative blood pressure will be different.

All patients are admitted to dedicated trauma wards and cared for in units that have written processes for administration of pre-operative fluids, choice of orthopaedic repair, and early mobilisation. All three units have policies mapped to national U.K. standards (National Institute for Health and Care Excellence [16], British Orthopaedic Association Standards for Trauma [17], Association of Anaesthetists of Great Britain and Ireland [18, 19] and Best Practice Tariff [20]).

This includes assessment by orthogeriatricians, operation within 36 hours of admission and assessment of bone health and falls. All patients are cared for under a hip fracture care pathway, which involves rapid assessment and admission from the emergency department, intravenous crystalloid infusions from the time of admission, and multi-professional care and discharge planning. Operations are performed in dedicated trauma theatres by appropriately experienced surgeons and anaesthetists.

In both groups, the attending anaesthetist will be responsible for provision of adequate analgesia and anaesthesia and appropriate post-operative fluid, haemoglobin and pain management. At a minimum, the following will be done:

- Haemoglobin concentrations will be maintained >80 g/L in both groups.
- Haemoglobin will be measured in the post-anaesthesia care unit using a point-of-care haemoglobin analyser (HemoCue™; Fisher Scientific, Pittsburgh, PA, USA) in all patients.
- Blood transfusion will be started in recovery if deemed clinically necessary.
- All patients will be encouraged to eat and drink as soon as possible following surgery.
- Regular paracetamol will be prescribed (dose adjusted if necessary).
- Rescue analgesia will be prescribed, avoiding non-steroidal anti-inflammatory drugs.

Surgical technique will be in line with standard national policies, though individual patient circumstances may require deviation from these on occasion, as follows:

- Displaced intra-capsular fracture: cemented hemiarthroplasty
- Extracapsular fracture: dynamic hip screw
- Sub-trochanteric fracture: dynamic hip screw or femoral nail

### Concomitant medication

Analgesia will be provided in accordance with normal practice at the study centre. Non-steroidal anti-inflammatory drugs are not routinely prescribed for this group of patients, owing to side effects. Other medications will be prescribed by the attending medical staff as appropriate for each individual. Thromboprophylaxis with subcutaneous low-molecular-weight heparin is routine.

### Concomitant treatments

Anaesthesia will be at the discretion of the attending anaesthetist; both spinal and general anaesthesia may be used. Peri-operative use of nerve blocks (femoral or fascia iliaca) is encouraged but not mandated in all of the units. Other post-operative therapies, such as physiotherapy, will be applied in accordance with routine hospital practice at the relevant centre.

### Study intervention

Patients randomised to standard blood pressure control will receive routine care. Fluids and vasopressor drugs will be used as deemed clinically appropriate by the attending anaesthetist.

Patients randomised to tight blood pressure control or avoidance of intra-operative hypotension will receive active treatment as required to maintain both of the following:

- Systolic arterial pressure >80% of baseline pre-operative value
- MAP >75 mmHg throughout

These targets have been purposefully set relatively high because we anticipate that, although there may be failure to achieve this target in all patients, intervention groups will be less likely to fall below thresholds with stronger associations with a poor outcome. The order and doses of treatments to achieve this will depend upon the clinical scenario but will include the following:

- Fluid bolus to ensure adequate intra-vascular volume (associated with reduced requirement for vasoactive drugs [11])
- Combined β-/α-1-adrenergic agonist (ephedrine)
- More selective α-1-adrenergic agonist (metaraminol)

Baseline pre-operative blood pressure will be defined as the mean of two readings taken at least 5 minutes apart after the patient has arrived in the induction room for anaesthesia. Patients will receive any anaesthetic technique deemed suitable by the attending anaesthetist in consultation with the patient and/or the patient’s relatives. Blood pressure will be measured using standard non-invasive equipment every 2.5 minutes or continuous non-invasive blood pressure monitoring in the intervention group. The frequency of blood pressure monitoring will be at the attending anaesthetist’s discretion in the control group, but at least every 5 minutes.

In addition to their normal care, each participant will be screened daily for the following:

- *Delirium using the 4 A’s Test (rapid assessment test for delirium) (4AT) tool*: The 4AT is a four-question screening tool for delirium that will be performed by either the ward nursing staff or a member of the research team.
- *Blood tests for renal function (including estimation of glomerular filtration rate) and troponin I*: These blood tests will be ordered with routine clinical care tests to reduce any additional burden for participants. Blood tests are typically ordered on 3 or 4 of the first 7 post-operative days.

A telephone follow-up call will be made to the patient (or their carer/close family member, or general practitioner [primary care physician] with appropriate consent) at 30 days to determine place of residence and functional status. Mortality status will be determined from administrative records at 1 year.

### Patient and process characteristics

Data will be recorded for pre-operative and intra-operative characteristics listed below.

#### Pre-operative

- Usual place of residence
- Date/time of admission
- Mobility prior to fracture
- Medical co-morbidities
- Frailty score [21]: 35-point version of Searle frailty index (excluding variables likely to be undefinable in patients with hip fracture)
- NHFS [15, 22–24]
- Full blood count
- Urea and electrolytes
- Arterial blood pressure (systolic, mean and diastolic)
  - Last ward measurement
  - Mean of two measurements 5 minutes apart in anaesthesia room before induction of anaesthesia

#### Intra-operative

- Timing/duration of surgery and anaesthesia
- Mode of anaesthesia and analgesia

### Outcome measures

Complications will be categorised according to European Perioperative Clinical Outcome (EPCO) definitions [25] as follows:

- Incidence of acute kidney injury in the first 7 days, defined as follows:
  - Stage 1: An increase in serum creatinine (sCr) of 1.5 to 1.99 times baseline *or* ≥0.3 mg/dl (≥26.5 μmol/L) increase
  - Stage 2: An increase in sCr of between 2 to 2.99 times baseline
  - Stage 3: An increase of three times or more baseline *or* increase in sCr to ≥4.0 mg/dl (≥353.6 μmol/L) after previous stage 1 *or* initiation of renal replacement therapy (a retrospective staging)
- Incidence of delirium in the first 7 days, defined using the AT4 [26, 27] delirium screening tool (This is a deliberate difference from EPCO due to specific evidence base for AT4 in hip fracture.)
- Incidence of myocardial injury after non-cardiac surgery in the first 3 days, defined as troponin >0.03 ng/ml judged to be due to myocardial injury in the absence of other causes
- Stroke: Embolic, thrombotic or haemorrhagic cerebral event with persistent residual motor, sensory or cognitive dysfunction [28]
- Length of hospital stay
- In-hospital mortality, 5- and 30-day mortality
- Quality of life recorded by EQ-5D [29] at 30 days
- 1-year mortality
- Ability to mobilise on 4-point scale compared with pre-morbid mobility

The ability of intervention group participants to achieve blood pressure limits will be assessed using a variety of methods:

- Nadir blood pressure (single lowest post-induction blood pressure)
- Absolute time below blood pressure limits
- Proportion of intra-operative time below blood pressure limits

### Statistical considerations

The primary outcome is a composite of presence or absence of defined cardiovascular, renal and delirium morbidity within 7 days of surgery:

- Myocardial injury following non-cardiac surgery *or*
- Stroke *or*
- Acute kidney injury *or*
- Delirium

Secondary outcomes are as follows:

- Presence or absence of each of the defined cardiovascular, renal and delirium morbidity within 7 days of surgery
- 5-day post-operative mortality
- 30-day post-operative mortality
- Operation to fit-for-discharge time
- Operation to up-and-walk time
- Proportion returning to pre-operative place of residence
- Prevalence of bone cement implantation syndrome [30]

The primary outcome is a composite binary outcome and will be analysed using chi-square statistics. Most of the supportive secondary outcomes are also binary outcomes and will be analysed similarly. Time data (e.g., length of stay) will be tested for normality. It is expected that reciprocal or logarithmic transformation will be required. Appropriate independent group tests (Student’s *t* or Mann-Whitney *U* test will be used). Mortality data will be analysed both as a binary outcome (30-day mortality, chi-square test) and using survival analysis (Cox regression). No formal interim analyses are planned.

### Sample size and justification

The incidence of acute kidney injury in patients with hip fracture is reportedly between 24% [12] and 45% [11]. Myocardial injury evidenced by elevated troponin occurs in about 27% of patients with hip fracture [31]. Delirium is variously reported. In our preliminary data (local pilot of 4AT tool), we found 20% patients had delirium. In a recent prospective study done in the United States in which researchers used the Confusion Assessment Method, an incidence of 33% of delirium was reported on post-operative day 2 [32]. In the recently reported neck of femur optimisation therapy - targeted stroke volume study (NOTTS) [11], which had a less robust collection of delirium data, researchers found that 56% of patients overall developed a cardiac, renal or neurological complication: 63% in the control group and 47% in the intervention group. A pessimistic assumption of a reduction in composite event rate from 50% to 45% would require a definitive trial size of around 4500 patients, accounting for a 10% drop-out rate.

Existing studies indicate an incidence of major organ dysfunction and major complications post-operatively in this population on the order of 20%. If tight blood pressure control peri-operatively decreased this incidence to 15% (a 25% improvement; similar to the effect size seen in NOTTS [11]), then 1250 patients would need to be randomised to each group to demonstrate this difference if the type I error rate were 5% and the type II error rate were 10% (power of 90%).

## Discussion

We are conducting a small-scale pilot feasibility study. Although there is mounting evidence of the association between intra-operative hypotension and worse outcome, there are few data demonstrating a causal link or that intervention is beneficial. Recent studies of cardiac output-guided fluid therapy in patients with hip fracture have provided inconclusive results [11, 33].

We have deliberately chosen a pragmatic intervention to manage intra-operative blood pressure. Hypotension during surgery and anaesthesia is multi-factorial, with a combination of previous and ongoing blood loss, dehydration, vasodilation (venous and arterial) and myocardial depression resulting from general or spinal anaesthesia, and concomitant drug therapy. The choice of fluids and vasoactive drugs for use in management is therefore dependent on the patient, the attending anaesthetist and the dynamic clinical situation. We therefore feel it is inappropriate, and also impractical, to protocolise how blood pressure is managed during anaesthesia.

The patients at greatest risk of complications following hip fracture are the frailer patients. Because this is a pilot study, patients who lack capacity are not being included. This therefore excludes the group that may have most to gain from the intervention [34]. We hope to address this in the main trial. As part of the pilot, we will be assessing the acceptability of the study to patients and their relatives. This information will inform the full trial design.

The study is unavoidably not blinded for the attending anaesthetist, and there is of course a risk that practice will drift towards the intervention practice. However, the high rates of hypotension observed during the ASAP study suggest that widespread ‘normal’ practice is not currently particularly effective at preventing hypotension. Intra-operative blood pressures are being recorded to assess the separation between groups.

There are myriad definitions of intra-operative hypotension. For this pilot study, we have chosen plausible, and we believe achievable, targets. Prior to a full study, these targets will be reassessed in light of information gained from the pilot study. Similarly, there is no single agreed definition of ‘normal’ blood pressure, particularly in the emergency patient, who may be affected by pain, anxiety, dehydration and hypovolaemia. The protocol explicitly allows for the attending anaesthetist to over-ride the anaesthesia room blood pressures (before the intervention starts) if these are felt to be non-representative.

We have chosen to limit the duration of the intervention to solely during anaesthesia, and not in the recovery or ward phases. Although hypotension in the recovery/post-anaesthesia care unit and the ward are anecdotally relatively common we felt it inappropriate to extend the intervention. This was for two reasons. First, in practical terms, during the period of anaesthesia and surgery, patients receive one-to-one care from a physician with the skills and resources to promptly and effectively prevent and treat cardiovascular disturbances. This is not the case in the ward, so an extended intervention period would not be practical outside a trial environment. Second, if the intervention is effective, then we anticipate that some of the causes of post-operative hypotension will have been mitigated before the post-operative period.

The anaesthetic and surgical episode is only a relatively brief period in the longer course of post-injury rehabilitation. Although it is possible that intra-operative management has no effect on longer-term outcome, the data derived from the ASAP study and other studies of intra-operative hypotension support the premise that it may have effects well beyond the immediate post-operative period. Improvements in hip fracture outcome have been associated with greater standardisation of surgical care and orthogeriatric care. Anaesthesia currently has relatively limited evidence on which to base standards [35]. The results of this study and the main trial would be expected to provide this evidence.

We have chosen a composite of four plausible, medically important complications. There is no good mechanistic reason to choose any one ahead of the others, and we therefore feel this is an appropriate approach. We hope that if this study shows positive effects, it will serve as a guide for larger studies investigating blood pressure control in both patients with hip fracture and more widely in older patients.

### Trial status

As of 14 July 2017, 22 of the planned 75 participants had been randomised.

## Additional files

Additional file 1: Figure S1. SPIRIT schedule of enrolment, interventions and assessments. (DOCX 78 kb)

Additional file 2: SPIRIT checklist. (DOCX 60 kb)

## Abbreviations

ASAP: Anaesthesia Sprint Audit of Practice
4AT: 4 A’s Test (rapid assessment test for delirium)
EPCO: European Perioperative Clinical Outcome
MAP: Mean arterial pressure
NHFS: Nottingham Hip Fracture Score
NOTTS: Neck of femur optimisation therapy - targeted stroke volume study
sCr: Serum creatinine
SPIRIT: Standard Protocol Items: Recommendations for Interventional Trials
