# Supplementary material for: Tight intra-operative blood pressure control versus standard care for patients undergoing hip fracture repair – Hip Fracture Intervention Study for Prevention of Hypotension (HIP-HOP) trial: study protocol for a randomised controlled trial
Source: Trials. 2017 Jul 25;18:350. doi: 10.1186/s13063-017-2066-5 (PMC5526232; doi:10.1186/s13063-017-2066-5)
Supplement: Supplementary file 1 — SPIRIT schedule of enrolment, interventions and assessments. (DOCX 78 kb) [file 13063_2017_2066_MOESM1_ESM.docx]

Figure S1. Schedule of enrolment, interventions, and assessments.

|  | ***STUDY PERIOD*** | | | | | | |
| --- | --- | --- | --- | --- | --- | --- | --- |
|  | **Enrollment** | **Allocation** | **Post-allocation** | | | | |
| **TIMEPOINT** | ***-t_1_*** | **0** | ***Intraoperative*** | ***Day_1-7_*** | ***Discharge*** | ***Day_30_*** | ***One year*** |
| **ENROLMENT:** |  |  |  |  |  |  |  |
| **Eligibility screen** | X |  |  |  |  |  |  |
| **Informed consent** | X |  |  |  |  |  |  |
| **Allocation** |  | X |  |  |  |  |  |
| **INTERVENTIONS:** |  |  |  |  |  |  |  |
| ***Tight blood pressure control*** |  |  | X |  |  |  |  |
| ***Standard care*** |  |  | X |  |  |  |  |
| **ASSESSMENTS:** |  |  |  |  |  |  |  |
| ***Pre-fracture information***  Residence, mobility, medical comorbidities  Nottingham Hip Fracture Score  Frailty Index  Full blood count, urea and electrolytes | X |  |  |  |  |  |  |
| ***Ward***  Pre-operative blood pressure | X |  |  |  |  |  |  |
| ***Anaesthetic room***  Blood pressure |  | X |  |  |  |  |  |
| ***Primary outcomes***  4AT scoring;  Troponin;  Serum creatinine  Clinical evidence of stroke / TIA |  |  |  | X |  |  |  |
| ***Other outcomes***  Medical complications |  |  |  | X |  |  |  |
| ***Intraoperative anaesthesia data***  Blood pressures  Mode of anaesthesia  Vasoactive drugs and fluids used  Occurrence of bone cement implantation syndrome |  |  | X |  |  |  |  |
| ***Post-operative course and discharge***  5-day postoperative mortality  operation to fit-for-discharge time  operation to up-and-walk time  mobility  discharge destination |  |  |  |  | X |  |  |
| ***30 day assessment***  Quality of life (EQ-5D)  30 day mortality |  |  |  |  |  | X |  |
| ***One year assessment***  One year mortality (from administrative data) |  |  |  |  |  |  | X |
